# Incidence and oncologic outcomes of patients with prostate‐specific antigen persistence after radical prostatectomy

**DOI:** 10.1002/cncr.70291

**Published:** 2026-02-05

**Authors:** Brian R. Lane, Patrick Lewicki, Sabrina L. Noyes, Corinne Labardee, Sabir Meah, Stephanie Daignault‐Newton, Jason Hafron, Todd M. Morgan, Archana Radhakrishnan, Robert T. Dess, James Montie, Khurshid R. Ghani, Kevin B. Ginsburg, Tudor Borza

**Affiliations:** ^1^ Division of Urology Corewell Health West Grand Rapids Michigan USA; ^2^ Department of Surgery Michigan State University College of Human Medicine Grand Rapids Michigan USA; ^3^ Department of Urology University of Michigan Ann Arbor Michigan USA; ^4^ Department of Urology Michigan Institute of Urology Troy Michigan USA; ^5^ Department of Urology Corewell Health East Royal Oak Michigan USA; ^6^ Oakland University William Beaumont School of Medicine Rochester Michigan USA; ^7^ Department of Internal Medicine University of Michigan Ann Arbor Michigan USA; ^8^ Department of Radiation Oncology University of Michigan Ann Arbor Michigan USA; ^9^ Division of Urology Wayne State University School of Medicine Detroit Michigan USA

**Keywords:** biochemical recurrence (BCR), diagnosis, management, prostate cancer

## Abstract

**Background:**

Patients with postradical prostatectomy (RP) prostate‐specific antigen (PSA) persistence (PPP) have been grouped with patients experiencing biochemical recurrence (BCR) in guidelines and clinical trials, potentially masking their distinct, unfavorable outcomes. The objective of this study was to determine whether patients with PPP constitute a unique, high‐risk population.

**Methods:**

The authors conducted a retrospective study of patients with prostate cancer undergoing RP (between January l, 2013 and June 30, 2024) using the Michigan Urological Surgery Improvement Collaborative (MUSIC) registry. Post‐RP status was defined as no evidence of disease (NED; never developed detectable PSA), PPP (first PSA remained detectable), and BCR (initially undetectable PSA with PSA ≥0.2 ng/mL later). The following differences were quantified: (1) pre‐RP characteristics, (2) pathologic characteristics at RP, (3) subsequent treatment patterns, and (4) differences in mortality outcomes based on postoperative PSA status.

**Results:**

Of 15,390 patients with who had a follow‐up of 4.0 years (interquartile range, 2.4–4.6 years), 11,019 still had NED, 1919 had PPP, and 2452 developed BCR. Patients who had PPP demonstrated a higher risk pre‐RP and post‐RP characteristics compared with those who had NED or BCR on unadjusted and adjusted comparisons, with differences that were both statistically significant and clinically meaningful. Patients who had PP had significantly higher PSA values before secondary treatment compared with those who had BCR (median PSA, 0.73 vs. 0.28 ng/mL, respectively; *p* < .001). The 5‐year all‐cause mortality rate was 2.5% (95% confidence interval, 2.1%–2.9%) for patients with initially undetectable PSA (NED and BCR combined) and 5.7% (95% confidence interval, 4.3%–5.7%) for patients with PPP (*p* < .001).

**Conclusions:**

One in eight patients who undergo prostatectomy experience PPP. These patients constitute a unique, high‐risk population distinct from patients who have NED and BCR. Clinical trials addressing optimal treatment and intensity for these at‐risk patients are critically warranted.

## Abstract 2

One in eight patients undergoing radical prostatectomy will never reach an undetectable prostate‐specific antigen level. These patients have more aggressive disease characteristics, substantially higher prostate‐specific antigen levels at secondary treatment, and worse overall mortality and thus constitute a unique, high‐risk population.

## INTRODUCTION

The oncologic status of patients undergoing radical prostatectomy (RP) for clinically localized prostate cancer (PCa) is largely determined by the post‐treatment prostate‐specific antigen (PSA) level.^1^ Undetectable post‐RP PSA stands as clinical evidence of treatment success and PSA recurrence (known as biochemical recurrence [BCR]) as evidence of primary treatment failure. The majority of guidelines and the evidence around post‐RP secondary treatment focus on BCR, which is defined by an initially undetectable postoperative PSA level followed by a relapse (typically, PSA ≥0.2 ng/mL on consecutive measurements).^2^ Nonetheless, a distinct, under‐recognized cohort of patients never achieve an undetectable PSA—that is, they have post‐RP PSA persistence (PPP)—comprising a state of persistent disease analogous to that managed by *consolidative therapy* (rather than adjuvant or salvage treatment) in other malignancies.^3^, ^4^

PPP is not a rare outcome after surgery, occurring in 3%–12% of patients.^5^ Although it has been hypothesized that PPP is clinically distinct from BCR, limited studies have reported the prevalence and prognostic significance of this potentially higher risk scenario, with heterogeneous results regarding its significance.^5^, ^6^, ^7^ Few prospective randomized controlled trials in the post‐RP space have drawn meaningful conclusions about optimal management, and some studies intended for patients with BCR have included those with PPP despite divergent characteristics based on assumptions that patients with early BCR and PPP would have similar high‐risk features.^8^, ^9^ Finally, clinical guidelines either do not distinguish PPP from BCR or provide only expert opinion recommendations for their management,^10^ reflecting the paucity of quality evidence.

In response to the inconsistent distinction in the literature between PPP and BCR, we test the hypothesis that patients who have PPP represent a distinct cohort with increased oncologic risk by comparing clinicopathologic characteristics and survival outcomes. We also seek to define the *real‐world* prevalence of PPP in a large quality‐improvement collaborative comprised of diverse patient populations and practice settings, and we shed light on a common, higher risk clinical outcome after RP that, to date, has largely been merged with BCR.

## MATERIALS AND METHODS

### Study population

We conducted a retrospective cohort study of patients with PCa who underwent RP using the prospectively maintained Michigan Urological Surgery Improvement Collaborative (MUSIC) registry. MUSIC is a quality‐improvement consortium involving over 260 urologists across 46 diverse practices in Michigan.^11^ We identified 23,117 men who underwent between January 1, 2013, and June 30, 2024 (see Figure S1). Men were excluded if they received any treatment before RP (neoadjuvant, *n* = 505) or after RP before the first PSA level was obtained (adjuvant, *n* = 749). We also excluded men who did not have a postoperative PSA level recorded or who had a detectable (>0.1 ng/mL) first PSA obtained before 35 days postoperatively and had no PSA repeated before 120 postoperative days because these patients with inadvertently obtained early PSA levels cannot be classified accurately (*n* = 2029). For men with undetectable PSA (≤0.1 ng/mL), we excluded those who had <1 year of follow‐up to more accurately distinguish the patients who developed BCR (*n* = 2452). Our final cohort included 15,390 patients.

### Study covariates

We classified each patient based on their postoperative PSA outcome. Patients were classified as having no evidence of disease (NED)—no residual or recurrent cancer—if the postoperative PSA remained undetectable(≤0.1 ng/mL) throughout study follow‐up. To create a more homogeneous *low‐risk* group, we required that patients who had NED never developed BCR, to facilitate the clinically meaningful comparative analyses between PPP and BCR. Patients were classified as having PPP if their first postoperative PSA was detectable—as defined by a PSA >0.1 ng/mL between days 35 and 120. Patients were classified as having BCR if they were had an initially undetectable postoperative PSA and then developed a PSA of 0.2 ng/mL or of 0.1 ng/mL followed by secondary treatment.^12^

For patients with PPP or BCR, we captured postoperative PSA values and time to secondary treatment, defined as the time between a PSA event (the date of first PSA among patients with PPP and the date of PSA indicating BCR among those with BCR) and the initiation of secondary treatment (either androgen‐deprivation therapy [ADT] or radiotherapy [RT]). Patients who had newly undetectable PSA without a record of secondary treatment were considered as having received secondary treatment, dated to the newly undetectable PSA, because it is highly improbable for PSA to go from detectable to undetectable without additional treatment. Each MUSIC practice obtained approval or exemption for collaborative participation from a local institutional review board.

### Study objectives and end points

The primary objective of this study was to evaluate whether patients who have PPP constitute a clinically distinct risk group compared with patients who have NED or develop BCR. To accomplish this, we quantified differences in four composite outcomes: (1) differences in pre‐RP characteristics, (2) differences in pathologic characteristics at RP, (3) subsequent treatment patterns for patients with PPP and BCR, and (4) differences in mortality outcomes.

### Statistical analysis

Clinicodemographic and oncologic characteristics were summarized as medians with interquartile ranges (IQRs) for continuous variables and counts with proportions for categorical variables. We performed univariate comparisons of characteristics between patients with NED and PPP or patients with BCR and PPP using Pearson χ^2^ tests for categorical variables and Wilcoxon rank‐sum tests for continuous variables. We performed adjusted analyses to define the association between clinicodemographic and oncologic characteristics with the development of PPP by using four separate multivariable logistic regression models; given the high degree of collinearity between prostate biopsy and RP characteristics, these variables were included in separate models. All models included a random effect for surgeon to account for intersurgeon correlation between procedures.

For patients with either PPP or BCR, we compared PSA parameters using Wilcoxon rank‐sum and Pearson χ^2^ tests. The time from PSA event to secondary treatment was compared using the Kaplan–Meier method, censoring patients at their last PSA level recorded in the registry. Again, two multivariable Cox regression models were used to evaluate associations between clinicopathologic characteristics and the time to secondary treatment, one for pre‐RP characteristics and one for RP characteristics, given the high degree of collinearity between prostate biopsy and RP characteristics. Both models included a random intercept for surgeon to account for inter‐surgeon correlation between procedures.

Finally, we evaluated differences in all‐cause mortality (ACM) and PCa‐specific mortality (PCSM) using the Kaplan–Meier method, censoring patients at their last entry in the registry. Death from causes other than PCa was considered a competing risk in the PCSM analysis. For ACM, we compared patients who had PPP with those who had an initially undetectable PSA after surgery (i.e., NED and BCR combined) and then separately with those who had NED and BCR separately. Given the low number of PCSM events and the clinical necessity of experiencing either PPP or BCR before PCa‐related mortality, we compared only patients who had PPP with those who had BCR. The log‐rank test and the Gray test were used to test for pairwise comparisons of survival curves for ACM and PCSM, respectively, and the Benjamini–Hochberg correction was used to account for multiple comparisons. For all time‐dependent analyses, we defined the at‐risk for event period as the date of first PSA for patients who had NED and PPP and the date of PSA event for those who had BCR to minimize risk of immortal person‐time bias.^13^

As a sensitivity analysis, we dichotomized the who had BCR into those experiencing early BCR (occurring within 1 year from RP) and late BCR (>1 year from RP), because it has been demonstrated that patients with early BCR have a higher risk of adverse oncologic events.^14^ By using methods similar to those used in the primary analyses, we defined the association between clinicodemographic and oncologic characteristics comparing patients who had PPP with these “highest risk” patients who had early BCR.

Statistical analysis was performed in R version 4.4.3 (R Foundation for Statistical Computing), with *p* values < .05 considered statistically significant. The R packages lme4 and coxme were used for model fitting, and the R ggsurvfit package was used to create Kapla–Meier curves.^15^, ^16^, ^17^

## RESULTS

Of the 15,390 patients, 11,019 had NED post‐RP throughout the length of their follow‐up, 1919 developed PPP, and 2452 developed BCR. The cohort had a median follow‐up of 4.0 years (IQR, 2.4–4.6 years). The median time to PPP event was 48 days (IQR, 41–62 days), and the median time to BCR event was 1.5 years (IQR, 0.75–2.6 years). Compared with patients who had NED or those BCR, patients who had PPP were more likely to have multiple known features of aggressive disease, and the magnitude of the differences was clinically meaningful (Table 1).

**TABLE 1:** Features of patients undergoing radical prostatectomy (RP) according to disease state: No evidence of disease, post‐RP prostate‐specific antigen persistence, or biochemical recurrence.

Characteristic	NED, *n* = 11,019	PPP, *n* = 1919	BCR, *n* = 2452	*p* for NED vs. PPP	*p* for BCR vs. PPP
Age: Median [IQR], years	64 [59–69]	65 [60–69]	65 [59–69]	< .001	.2
Race				< .001	< .001
White	8439 (77.0)	1361 (71.0)	1910 (78.0)		
African American	1275 (12.0)	296 (15.0)	301 (12.0)		
Other	279 (2.5)	49 (2.6)	51 (2.1)		
Unknown	1026 (9.3)	213 (11.0)	190 (7.7)		
Charlson score				.042	.3
0	8209 (75.0)	1381 (72.0)	1803 (74.0)		
1	1738 (16.0)	344 (18.0)	396 (16.0)		
2	1071 (9.7)	193 (10.0)	253 (10.0)		
BMI: Median [IQR], kg/m^2^	28.7 [25.9–31.7]	28.9 [25.8–32.3]	28.9 [26.1–32.3]	.2	.3
Clinical tumor (cT) stage				< .001	< .001
cT1	7960 (72.0)	1054 (55.0)	1506 (62.0)		
cT2	2054 (19.0)	585 (31.0)	699 (29.0)		
cT3	79 (0.7)	75 (3.9)	44 (1.8)		
cTx	895 (8.1)	197 (10.0)	198 (8.1)		
Preoperative PSA: Median [IQR], ng/mL	6.2 [4.8–8.5]	9.6 [6.4–16.5]	7.3 [5.4–11.2]	< .001	< .001
Preoperative PSA group, ng/mL				< .001	< .001
<10	8973 (83.0)	981 (52.0)	1665 (69.0)		
10–20	1574 (14.0)	538 (29.0)	552 (23.0)		
20–50	295 (2.7)	283 (15.0)	170 (7.0)		
>50	33 (0.3)	77 (4.1)	30 (1.2)		
Biopsy ISUP GG				< .001	< .001
GG1	2170 (20.0)	145 (7.6.0)	218 (8.9)		
GG2	5514 (50.0)	437 (23.0)	843 (35.0)		
GG3	2194 (20.0)	562 (29.0)	692 (28.0)		
GG4	816 (7.4)	411 (22.0)	427 (17.0)		
GG5	283 (2.6)	354 (19.0)	263 (11.0)		
NCCN risk group				< .001	< .001
Very low	306 (2.8)	13 (0.7)	20 (0.8)		
Low	1537 (14.0)	95 (5.0)	147 (6.0)		
Favorable intermediate	3223 (29.0)	183 (9.6)	391 (16.0)		
Unfavorable intermediate	4558 (42.0)	676 (35.0)	1084 (44.0)		
High	764 (7.0)	335 (18.0)	294 (12.0)		
Very high	579 (5.3)	608 (32.0)	507 (21.0)		
Preoperative MRI	3203 (29.0)	644 (34.0)	759 (31.0)		
RP year category				< .001	< .001
Before 2020	7416 (67.0)	1144 (62.0)	1714 (70.0)		
2020	983 (8.9)	134 (7.3.0)	238 (9.7)		
After 2020	2620 (24.0)	554 (30.0)	496 (20.0)		
Prostatectomy GG				< .001	< .001
GG1	1309 (12.0)	63 (3.3)	87 (3.6.0)		
GG2	6664 (61.0)	459 (24.0)	950 (39.0)		
GG3	2243 (21.0)	630 (33.0)	841 (35.0)		
GG4	338 (3.1)	219 (11.0)	189 (7.8)		
GG5	362 (3.3)	538 (28.0)	369 (15.0)		
Prostatectomy pathologic tumor (pT) stage				< .001	<.001
pT2	7994 (7.0)	521 (27.0)	1093 (45.0)		
pT3a	2549 (23.0)	674 (35.0)	906 (37.0)		
pT3b	450 (4.1)	694 (36.0)	448 (18.0)		
pT4	3 (<0.1)	27 (1.4)	4 (0.2)		
Prostatectomy pathologic lymph node (pN) stage				< .001	< .001
pN0	8295 (75.0)	1337 (70.0)	1972 (80.0)		
pN1	111 (1.0)	351 (18.0)	128 (5.2.0)		
pNx	2613 (24.0)	231 (12.0)	352 (14.0)		
Positive surgical margins	2929 (27.0)	1124 (59.0)	1215 (50.0)	< .001	< .001
Adverse pathology: GG4/GG5 and T3/T4	325 (2.9)	662 (34.0)	399 (16.0)	< .001	< .001

*Note*: Data were missing for Charlson score (*n* = 2), clinical tumor stage (*n* = 44), PSA (*n* = 219), biopsy GG (*n* = 61), NCCN risk group (*n* = 70), prostatectomy GG (*n* = 129), and prostatectomy T stage (*n* = 27).

Abbreviations: BCR, biochemical recurrence; BMI, body mass index; GG, grade group; IQR, interquartile range; ISUP, International Society of Urological Pathology; MRI, magnetic resonance imaging; NCCN, National Comprehensive Cancer Network; NED, no evidence of disease; PPP, post‐RP prostate‐specific antigen persistence; PSA, prostate‐specific antigen.

The presence of known high‐risk features was significantly associated with patients who had PPP in adjusted analyses compared with those who had NED (see Table S1). Compared with patients who had BCR, those who had higher PSA (odds ratio [OR], 1.74 per log ng/mL; 95% confidence interval [CI], 1.58–1.92 per log ng/mL), higher clinical T (cT) stage (cT3 vs. cT1: OR, 1.59; 95% CI, 1.04–2.42; *p* = .031), higher biopsy grade group (GG; GG5 vs. GG1: OR, 1.64; 95% CI, 1.23–2.19), and surgery date after 2020 (OR, 1.61 vs. before 2020; 95% CI, 1.37–1.89) were associated with PPP in the pre‐RP model; whereas higher PSA (OR, 1.55 per log ng/mL; 95% CI, 1.39–1.72 per log ng/mL), higher pathologic T (pT) stage (pT3b vs. pT2: OR, 1.95; 95% CI, 1.60–2.37), the presence of nodal disease (OR, 2.80; 95% CI, 2.20–3.57), and surgery date after 2020 (OR, 1.60 vs. before 2020; 95% CI, 1.36 per log ng/mL 1.89) had statistically significant associations with PPP in the post‐RP model (Table 2; *p* < .001 for all unless mentioned). Notably, positive surgical margins (*p* = .3) and the presence of GG4 or GG5 disease on final pathology (*p* = .7 for both) were not associated with PPP in this analysis.

**TABLE 2:** Model identifying factors associated with postradical prostatectomy prostate‐specific antigen persistence compared with to biochemical recurrence after radical prostatectomy.

Characteristic	Odds ratio	95% confidence interval	*p*
Pre‐RP model			
Age (per 5 years)	1.00	0.96–1.05	.9
Race (Ref, White)			.019^a^
African American	1.15	0.94–1.40	.2
Other	1.08	0.70–1.66	.7
Unknown	1.45	1.14–1.85	.003^a^
Charlson score (Ref, 0)			.4
1	1.10	0.92–1.31	.3
≥2	0.93	0.75–1.16	.5
Log preoperative PSA	1.74	1.58–1.92	< .001^a^
RP year category (Ref, before 2020)			< .001^a^
2020	0.87	0.68–1.11	.3
After 2020	1.61	1.37–1.89	< .001^a^
Biopsy GG (Ref, GG1)			< .001^a^
GG2	0.68	0.52–0.88	.003^a^
GG3	0.98	0.76–1.27	.9
GG4	1.15	0.88–1.51	.3
GG5	1.64	1.23–2.19	< .001^a^
Clinical tumor (cT) stage (Ref, cT1)			.006^a^
cT2	1.13	0.97–1.31	.11
cT3	1.59	1.04–2.42	.031^a^
cTx	1.39	1.10–1.76	.005^a^
Post‐RP model			
Age (per 5 years)	0.97	0.92–1.02	.2
Race (Ref, White)			.007^a^
African American	1.17	0.95–1.44	.15
Other	1.13	0.73–1.76	.6
Unknown	1.53	1.19–1.97	< .001^a^
Charlson score (Ref, 0)			.4
1	1.05	0.88–1.26	.6
≥2	0.88	0.70–1.11	.3
Log preoperative PSA	1.55	1.39–1.72	< .001^a^
RP year category (Ref, before 2020)			< .001^a^
2020	0.88	0.68–1.13	.3
After 2020	1.60	1.36–1.89	< .001^a^
Surgical GG (Ref, GG1)			< .001^a^
GG2	0.52	0.36–0.75	< .001^a^
GG3	0.69	0.47–1.01	.058
GG4	1.09	0.71–1.67	.7
GG5	1.09	0.73–1.64	.7
Positive surgical margins	1.08	0.94–1.24	.3
Pathologic tumor (pT) stage (Ref, pT2)			< .001^a^
pT3a	1.24	1.05–1.47	.013^a^
pT3b	1.95	1.60–2.37	< .001^a^
pT4	7.49	2.48–22.6	< .001^a^
Pathologic lymph node (pN) stage (Ref, pN0)			< .001^a^
pN1	2.80	2.20–3.57	< .001^a^
pNx	1.32	1.07–1.64	.011^a^

Abbreviations: GG, grade group; PPP, postradical prostatectomy prostate‐specific antigen persistence; PSA, prostate‐specific antigen; Ref, reference category; RP, radical prostatectomy.

^a^ These *p* values indicate statistical significance.

**FIGURE 1:**
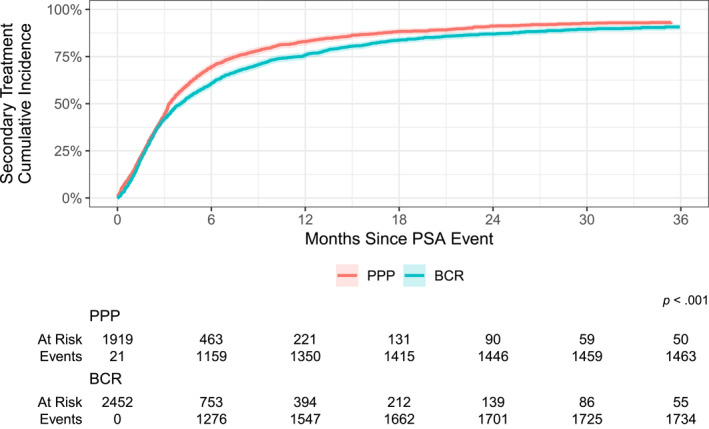
Time to secondary treatment by disease status (log‐rank *p* < .001). BCR indicates biochemical recurrence; PPP, postradical prostatectomy prostate‐specific antigen persistence; PSA, prostate‐specific antigen.

**FIGURE 2:**
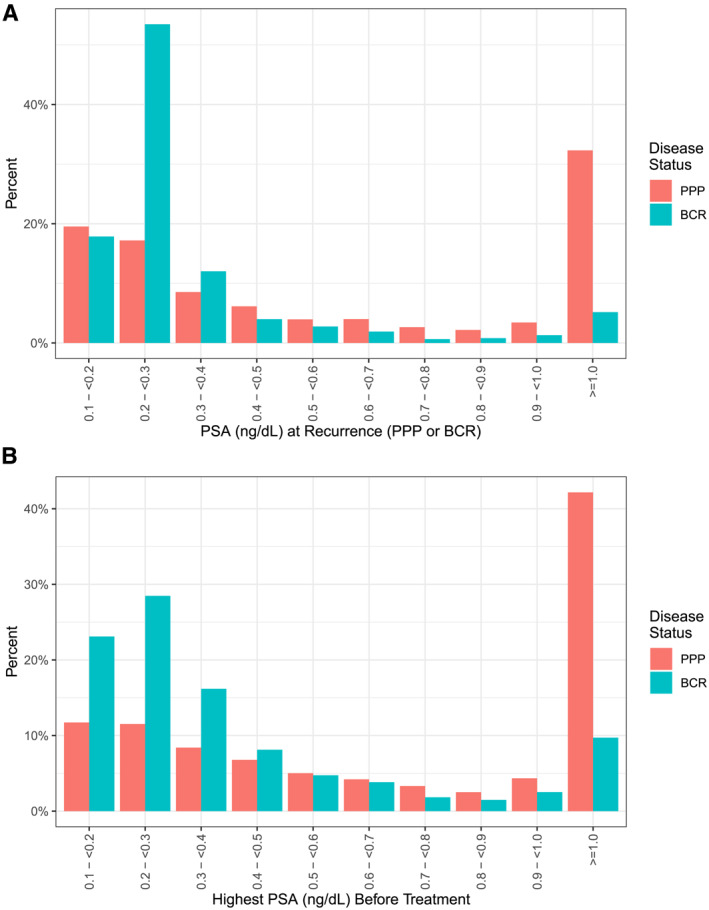
Post‐RP PSA values at PSA event, stratified by disease status, for (A) all patients experiencing PPP (*n* = 1919; red bars) or BCR (*n* = 2452; blue bars) and (B) those undergoing secondary treatment for PPP (*n* = 1475; red bars) or BCR (*n* = 1749; blue bars). BCR indicates biochemical recurrence; PPP, postradical prostatectomy prostate‐specific antigen persistence; PSA, prostate‐specific antigen; RP, radical prostatectomy.

Although >90% of patients with either PPP or BCR received secondary treatment within 3 years of their PSA event, patients with PPP were treated significantly sooner than those with BCR (Figure 1; Kaplan–Meier estimated 6‐month treatment rate, 70% vs. 61%; *p* < .001). Patients who had PPP had significantly higher PSA levels compared with those who had BCR at the time of PSA event (0.45 vs. 0.20 ng/mL; *p* < .001); 49% of patients with PPP had a PSA >0.5 ng/mL, and 32% had a PSA >1.0 ng/mL compared with 13% and 4.8%, respectively, among those with BCR (Figure 2). Among patients who received secondary treatment for PPP or BCR, the median number of PSA tests before secondary treatment was two (IQR, from one to three tests). The PSA level before secondary treatment was significantly higher for patients who had PPP (median PSA, 0.73 vs. 0.28 ng/mL; *p* < .001), with 38% receiving treatment at a PSA level <0.5 ng/mL compared with 76% of patients who had BCR.

**FIGURE 3:**
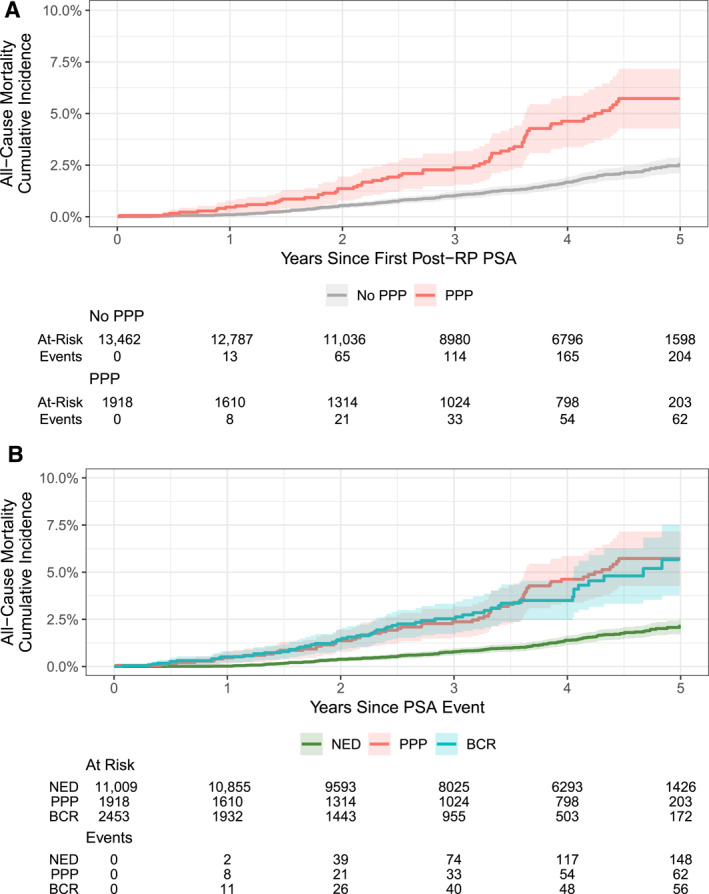
Cumulative incidence of all‐cause mortality by disease status. Time 0 is first the postoperative PSA level for patients with NED and PPP and PSA‐defining BCR for those with BCR. (A) Log‐rank *p* < .001 for all‐cause mortality after the first postoperative PSA level. (B) Pairwise log‐rank *p* < .001 for both PPP and BCR versus NED and *p* = .4 for those with PPP versus BCR for all‐cause mortality after a PSA event defining PPP or BCR. BCR indicates biochemical recurrence; NED, no evidence of disease; PPP, postradical prostatectomy prostate‐specific antigen persistence; PSA, prostate‐specific antigen; RP, radical prostatectomy.

We observed 266 deaths, none which were prostate‐cancer specific, at a median follow‐up of 4.3 years (IQR, 2.9–4.7 years), among patients with NED; 74 deaths, with 17 caused by PCa, at a median follow‐up of 3.3 years from PSA event date (IQR, 1.6–4.6 years), among patients with PPP; and 57 deaths, with seven caused by PCa, at a median follow‐up of 2.4 years from PSA event date (IQR, 1.2–3.7 years), among patients with BCR. The cumulative incidence of ACM at 5 years after RP was 2.5% (95% CI, 2.1%–2.9%) for patients with initially undetectable PSA (i.e., NED and BCR combined) and 5.7% (95% CI, 4.%3–5.7%) for patients with PPP (*p* < .001; Figure 3A). ACM was significantly higher for patients who had either PPP or BCR compared with those who had NED (*p* < .001 for both pairwise comparisons; Figure 3B), whereas no significant difference in mortality after PSA event was observed between patients who had PPP versus those who had BCR (*p* = .4). A nonstatistically significant difference was noted in PCSM among patients who had PPP compared with those who had BCR (1.3% vs. 1.1% at 5 years; *p* = .073; see Figure S2).

In our sensitivity analysis, among the patients who had BCR, we classified 854 (35%) with early BCR and 1598 (65%) with late BCR (see Table S2). The results of the comparison between patients who had PPP and the *highest risk* early BCR group largely confirm the findings of the primary analysis in patients with PPP, demonstrating a higher incidence of most high‐risk features in unadjusted analyses. On adjusted analyses, higher odds of PPP versus early BCR were associated with higher preoperative PSA (OR, 1.40; *p* < .001), higher prostatectomy tumor stage (T3b vs. T2: OR, 1.52; *p* = .001; T4 vs. T2: OR, 4.45; *p* = .018), nodal disease (N1 vs. N0: OR, 2.35; *p* < .001), and less frequent positive surgical margins (OR, 0.76; *p* = .004).

## DISCUSSION

Greater than 10% of patients who undergo RP have persistently positive PSA levels and can be categorized as a distinctly high‐risk cohort that, to date, has been inadequately distinguished from patients who have typical BCR. Here, we demonstrate that, in a large, statewide collaborative capturing more than 15,000 patients undergoing RP, those with PPP have significantly worse pre‐RP and post‐RP clinicopathologic characteristics compared with patients who have NED or BCR and, despite a short time from PSA event to treatment, they also have higher ACM than patients with initially undetectable PSA, including notable rates of early PCSM. Our results highlight the need for further dedicated study of this population to better define long‐term oncologic outcomes and inform initial treatment selection and secondary treatment intensity. The timeliness of such research is made apparent by our finding of the increasing prevalence of PPP in recent years, which accords with other studies demonstrating an increasing biologic risk among patients undergoing surgery over time.^18^

Currently, there is no clear guidance for and a paucity of high‐quality evidence on the optimal management of patients with PPP. This is partially attributed to the inconsistent distinction of patients with PPP from those with BCR. The American Urological Association guideline on salvage therapy for PCa lists risk factors for consideration of salvage treatment at a PSA <0.2 ng/dL but lumps PPP with short‐interval BCR and other factors of varying significance.^2^ The European Association of Urology guideline does distinguish PPP from BCR but gives treatment recommendations a *weak* strength of evidence rating, given the sparse to nonexistent trial‐level evidence for this clinical scenario.^10^ Our findings demonstrate that patients who have PPP harbor more aggressive clinical and pathologic features compared with those who have BCR, even when that comparison is made with patients who have early BCR, as demonstrated in our sensitivity analysis, raising questions about the accuracy of these guidelines. Recent evidence in the post‐RP management space comes from a pair of randomized trials comparing adjuvant versus early salvage treatment for patients at elevated risk of post‐RP BCR (RAVES^19^ [ClinicalTrials.gov identifier NCT00860652] and RADICALS‐RT^20^ [ClinicalTrials.gov identifier NCT00544147]). RAVES excluded patients who met our definition of PPP, whereas RADICALS‐RT included patients with an initial PSA ≤0.2 ng/dL—which represents <20% of patients with PPP because most present with significantly higher PSA levels based on our data—thus, neither study can be extended confidently to patients who have PPP.

For patients with a suspicion of disease recurrence after RP, secondary treatment is typically categorized as either *adjuvant* or *salvage* (in the absence or presence of detectable disease, respectively). These categories may not adequately address the management and treatment intensification of patients with PPP, which draws its own analogy to *consolidative* treatment used in other malignancies.^3^, ^4^, ^21^, ^22^ Consolidative treatment, directed toward known residual disease rather than prevention in an at‐risk but yet‐to‐recur population, features in the management of nonsmall cell lung cancer,^3^ leukemia,^21^ and numerous other malignancies.^22^, ^23^ Optimal consolidative treatment for PPP is unknown. Based on the outcomes of the RAVES and RADICALS‐RT trials, guidelines recommend treatment below a PSA of 0.5 ng/mL, with increasing mortality occurring above this threshold.^12^ In our current study, over one half of patients with PPP had a PSA that exceeded this threshold at diagnosis, and nearly one third had a PSA >1 ng/mL, raising concern that these patients could have a substantially poorer response to the evaluated secondary treatments.

Several findings among patients with PPP, including the high PSA levels at diagnosis, the high proportion with pathologic N1 disease (18%), and the higher incidence of ACM and occurrence of PCSM within a relatively short follow‐up timeframe indicate that systemic treatment, rather than localized approaches alone, should be strongly considered for these patients, aligned with the guideline‐recommended addition of ADT for patients who have BCR and the presence of high‐risk pathologic features. However, the addition of short‐term ADT may not be adequate given the multiple high‐risk features present in many patients with PPP, and treatment considerations are likely to diverge from those of typical BCR in which the current management paradigm provides local treatment (early salvage RT) while attempting to minimize unnecessary systemic treatment, whereas recurrent disease is still likely to be local. Recent advances in the management of advanced PCa offer myriad approaches to treatment escalation, including the addition of androgen receptor inhibitors to traditional ADT, the use of immune‐oncology or mutation‐directed agents, and the upfront use of traditional chemotherapy.^24^ The degree to which any of these treatments are appropriate remains unknown; regardless, multidisciplinary management of these patients is imperative. Ultimately, the increased adoption of prostate‐specific membrane antigen (PSMA) positron emission tomography (PET) testing, with its increased detection of extraprostatic disease compared with conventional imaging, should better inform management both in terms of pre‐RP selection and post‐RP treatment escalation.^25^

Our findings accord with and elaborate on previous reports describing patients with PPP. Ploussard et al. conducted a systematic review on the management of PSA persistence, highlighting the heterogeneity of cohorts and definitions across several single‐center and/or small series.^5^ Tilki et al. and Preisser et al. describe similarly high‐risk features and poor outcomes in tertiary‐center and cancer registry data in Europe, respectively.^6^, ^26^ More recently, in an analysis of two academic cohorts, Tilki et al. describe findings similar to ours, including increased incidence of high‐risk clinical features and increased mortality among patients with PPP.^6^ Our 12% incidence of PPP is at the high end of their reported range and may more accurately represent real‐world practice outcomes because we uniquely estimate the prevalence of PPP in a large US cohort that spans diverse patient populations and practice types. Our results also demonstrate a temporal increase in the prevalence of PPP, which may reflect changing practice patterns with increasing active surveillance rates for favorable‐risk PCa and increasing operative management of high‐risk localized, locally advanced, and oligometastatic PCa.^27^

Finally, we noted an increased incidence of ACM for patients who had PPP compared with those who had initially undetectable PSA, although this difference did not persist when comparing patients who had PPP with those who only had BCR. We believe that a significant proportion of ACM is indeed driven by PCSM, which is difficult to consistently attribute given registry design. Our nuanced approach to this analysis highlights the challenges with interpreting comparative mortality risks from existing studies, which frequently combine all patients who have undetectable PSA, even if they later progress to BCR, resulting in heterogenous cohorts and ambiguous risk estimates. Notably, the presence of mortality events, although rare, in the first 5 years after prostatectomy speaks to the high‐risk nature of our cohort, among both patients with PPP and those with BCR, and is consistent with mortality rates noted in other high‐risk prostatectomy cohorts (e.g., patients with pathologic N1 disease).^28^ Parallels between post‐RP PSA persistence and post‐RT PSA nadir, which is an important predictor of PCa metastasis and downstream outcomes, are apparent.^29^ Despite the lack of an ACM difference between PPP and BCR after PSA event, because PPP occurred on average 1.5 years earlier than BCR in our study, mortality events so early after prostatectomy may point to an opportunity for improved patient selection.

Our findings should be considered in the context of several limitations. First, our study leverages the robust data‐abstraction infrastructure at the individual urology practice level of a mature, statewide quality‐improvement collaborative; however, despite this, it is possible that our prospective database under‐captures mortality events. Second, MUSIC follows patients for 5 years from treatment, limiting our ability to capture events that occur outside this timeframe. Furthermore, metastasis is not uniformly included in our registry. This limitation is somewhat mitigated by our high‐fidelity, granular clinical and pathologic data at the time of diagnosis and initial treatment that allows for accurate distinction between patients with NED, PPP, and BCR and facilitates risk assessment in this population. In addition, the presence of early mortality events speaks to the high‐risk nature of patients who have PPP and warrants dedicated future investigation. Third, details on additional treatment(s) received (e.g., extent of radiation, receipt/duration of ADT) are incomplete because these are frequently performed outside urology practices, limiting access of the data abstractors to these details. We suspect that treatment intensity is greater amongst patients who have PPP, further emphasizing the significance of observed differences in PCSM cumulative incidence between PPP and BCR. Similarly, data on ultrasensitive PSA tests (those reporting values <0.1 ng/mL) are not captured in the registry. Last, most patients in our study were treated before the widespread adoption of staging PSMA‐PET. PSMA‐PET was not captured in the MUSIC registry until July 2023; therefore, it is impossible to provide an exact rate among the study cohort. We estimate that, in 2024, approximately 50% of National Comprehensive Cancer Network high‐risk patients and 20% of unfavorable intermediate‐risk patients were staged with PSMA‐PET. Although real‐world data on the ability of PSMA‐PET to correctly discriminate patients with potential low‐volume extraprostatic disease remain unclear, we acknowledge a clear role for PSMA‐PET in both the preoperative and postoperative management of these patients; therefore, our findings may be limited in their generalizability to contemporary cohorts.

## CONCLUSIONS

We describe the population‐level prevalence and clinical and prognostic significance of an under‐recognized, clinically distinct, high‐risk subset of patients with PCa. These results highlight the need for dedicated prospective study and clinical trials oriented at patients with PPP, distinct from BCR, to inform optimal management.

## AUTHOR CONTRIBUTIONS

**Brian R. Lane**: Conceptualization; methodology; data curation; writing—review and editing; supervision; resources; funding acquisition. **Patrick Lewicki**: Methodology; data curation; writing—original draft; writing—review and editing. **Sabrina L. Noyes**: Data curation; investigation; writing—review and editing; resources. **Corinne Labardee**: Resources; project administration. **Sabir Meah**: Software; formal analysis; visualization. **Stephanie Daignault‐Newton**: Visualization; software; formal analysis; writing—review and editing. **Jason Hafron**: Data curation; writing—review and editing. **Todd M. Morgan**: Data curation; writing—review and editing. **Archana Radhakrishnan**: Writing—review and editing. **Robert T. Dess**: Data curation; writing—review and editing. **James Montie**: Writing—review and editing; supervision. **Khurshid R. Ghani**: Data curation, writing—review and editing; funding acquisition. **Kevin B. Ginsburg**: Conceptualization; methodology; data curation; writing—review and editing. **Tudor Borza**: Conceptualization; methodology; data curation; writing—review and editing; supervision; funding acquisition.

## CONFLICT OF INTEREST STATEMENT

Jason Hafrom reports personal/consulting fees from ArteraAI, Astellas Pharma US Inc., Dendreon Pharmaceuticals LLC, Janssen Biotech Inc., Myriad Genetics Inc., Novartis Pharmaceuticals Corporation, Pfizer Inc., and Photocure Inc.; and support for other professional activities from AstraZeneca Pharmaceuticals LP, Bayer HealthCare Pharmaceuticals Inc., Immunis.AI:Scientific, Merck & Company Inc., and Sumitomo Dainippon Pharma Oncology outside the submitted work. Todd M. Morgan reports personal/consulting fees from Merck & Company Inc., Pfizer Inc., and Foundation Medicine Inc. outside the submitted work. The remaining authors declared no conflicts of interest.

## Supporting information

Supplementary Material

Supplementary Material

Supplementary Material

Supplementary Material

Supplementary Material

## Data Availability

Research data are not shared.
