# Supplementary material for: Incidence and oncologic outcomes of patients with prostate‐specific antigen persistence after radical prostatectomy
Source: Cancer. 2026 Feb 5;132(4):e70291. doi: 10.1002/cncr.70291 (PMC12876554; doi:10.1002/cncr.70291)
Supplement: Supplementary file 1 — Supplementary Material [file CNCR-132-e70291-s003.docx]

**FIGURE S1**. CONSORT diagram

**FIGURE S2.** Cumulative incidence of prostate cancer (PCa) specific mortality by disease status. Time 0 is first postoperative PSA for PPP, and PSA defining BCR for BCR. Log-rank *p* = .073.
