# Supplementary figures and images for: Incidence and oncologic outcomes of patients with prostate‐specific antigen persistence after radical prostatectomy
Source: Cancer. 2026 Feb 5;132(4):e70291. doi: 10.1002/cncr.70291 (PMC12876554; doi:10.1002/cncr.70291)

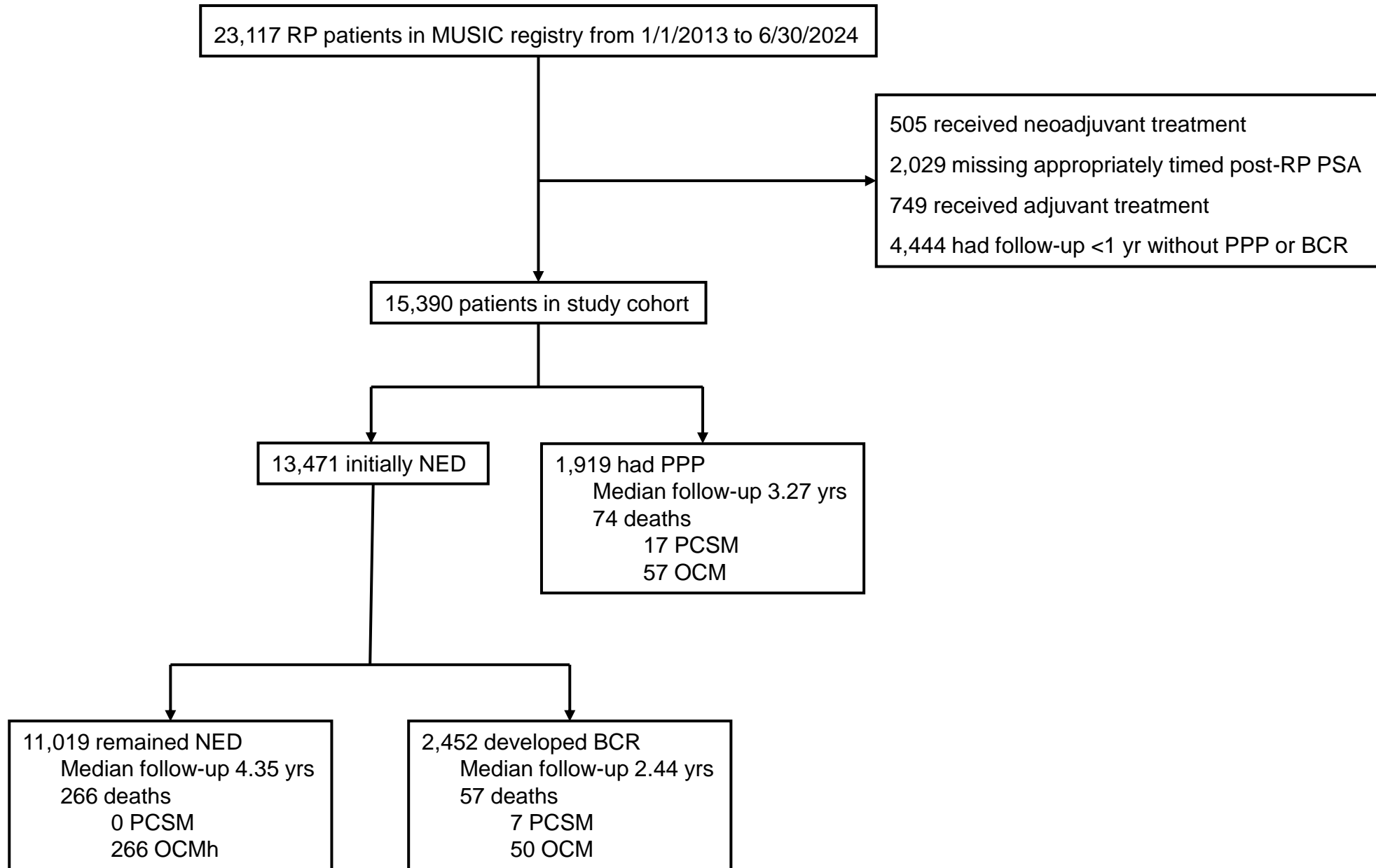

Supplement: Supplementary file 2 — Supplementary Material [file CNCR-132-e70291-s002.pdf]

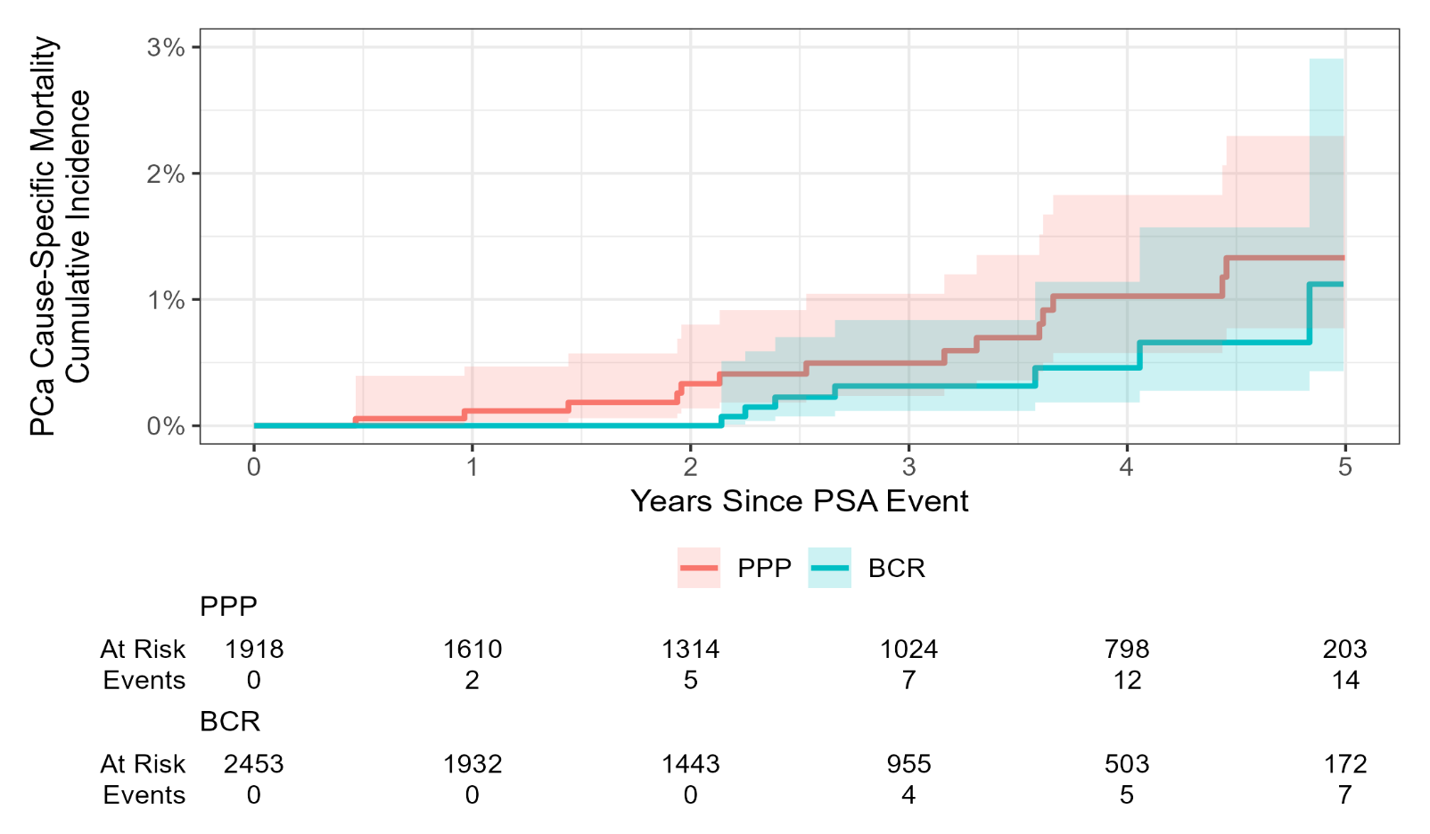

Supplement: Supplementary file 3 — Supplementary Material [file CNCR-132-e70291-s005.tif]
