# Supplementary material for: Incidence and oncologic outcomes of patients with prostate‐specific antigen persistence after radical prostatectomy
Source: Cancer. 2026 Feb 5;132(4):e70291. doi: 10.1002/cncr.70291 (PMC12876554; doi:10.1002/cncr.70291)
Supplement: Supplementary file 4 — Supplementary Material [file CNCR-132-e70291-s001.docx]

**Table S1.** Model identifying factors associated with persistently positive PSA compared to no evidence of recurrence.

| Characteristic | Odds Ratio | 95% Confidence Interval | p-value |
| --- | --- | --- | --- |
| Pre-Prostatectomy Model | | | |
| Age (Per 5 Years) | 0.96 | 0.92, 1.00 | **0.046** |
| Race (Ref: White) |  |  | **0.018** |
| African American | 1.04 | 0.87, 1.24 | 0.7 |
| Other | 0.77 | 0.53, 1.12 | 0.2 |
| Unknown | 1.35 | 1.09, 1.67 | **0.006** |
| Charlson Score (Ref: 0) |  |  | 0.3 |
| 1 | 1.09 | 0.94, 1.28 | 0.3 |
| ≥2 | 0.93 | 0.76, 1.13 | 0.5 |
| Log Pre-Operative PSA | 3.10 | 2.82, 3.40 | **<0.001** |
| RP Year Category (Ref: Pre-2020) |  |  | **0.005** |
| 2020 | 0.83 | 0.66, 1.03 | 0.092 |
| Post-2020 | 1.19 | 1.03, 1.37 | **0.019** |
| Biopsy GG (Ref: GG1) |  |  | **<0.001** |
| GG2 | 1.12 | 0.90, 1.38 | 0.3 |
| GG3 | 3.18 | 2.57, 3.94 | **<0.001** |
| GG4 | 5.96 | 4.73, 7.50 | **<0.001** |
| GG5 | 14.3 | 11.0, 18.5 | **<0.001** |
| Clinical T-Stage (Ref: cT1) |  |  | **<0.001** |
| cT2 | 1.78 | 1.56, 2.04 | **<0.001** |
| cT3 | 3.33 | 2.19, 5.08 | **<0.001** |
| cTx | 1.59 | 1.30, 1.93 | **<0.001** |
| Post-Prostatectomy Model | | | |
| Age (Per 5 Years) | 0.89 | 0.85, 0.93 | **<0.001** |
| Race (Ref: White) |  |  | **0.008** |
| African American | 1.06 | 0.86, 1.30 | 0.6 |
| Other | 0.74 | 0.49, 1.11 | 0.14 |
| Unknown | 1.43 | 1.13, 1.80 | **0.003** |
| Charlson Score (Ref: 0) |  |  | 0.3 |
| 1 | 1.04 | 0.87, 1.23 | 0.7 |
| >=2 | 0.86 | 0.68, 1.07 | 0.2 |
| Log Pre-Operative PSA | 2.20 | 1.98, 2.45 | **<0.001** |
| RP Year Category (Ref: Pre-2020) |  |  | 0.12 |
| 2020 | 0.77 | 0.60, 0.99 | 0.038 |
| Post-2020 | 0.97 | 0.83, 1.14 | 0.7 |
| Prostatectomy GG (Ref: GG1) |  |  | **<0.001** |
| GG2 | 0.91 | 0.67, 1.23 | 0.5 |
| GG3 | 2.47 | 1.81, 3.37 | **<0.001** |
| GG4 | 6.25 | 4.37, 8.94 | **<0.001** |
| GG5 | 8.77 | 6.22, 12.4 | **<0.001** |
| Positive Surgical Margins | 2.41 | 2.11, 2.76 | **<0.001** |
| Pathological T-Stage (Ref: pT2) |  |  | **<0.001** |
| pT3a | 2.10 | 1.80, 2.44 | **<0.001** |
| pT3b | 6.29 | 5.18, 7.63 | **<0.001** |
| pT4 | 22.4 | 5.78, 86.8 | **<0.001** |
| Pathological N-Stage (Ref: pN0) |  |  | **<0.001** |
| N1 | 5.81 | 4.37, 7.74 | **<0.001** |
| Nx | 1.09 | 0.90, 1.33 | 0.4 |

Abbreviations: GG, grade group
