# Supplementary material for: Incidence and oncologic outcomes of patients with prostate‐specific antigen persistence after radical prostatectomy
Source: Cancer. 2026 Feb 5;132(4):e70291. doi: 10.1002/cncr.70291 (PMC12876554; doi:10.1002/cncr.70291)
Supplement: Supplementary file 5 — Supplementary Material [file CNCR-132-e70291-s004.docx]

**TABLE S2.** Features of patients undergoing RP according to disease state: PPP, or early BCR (within 12 months from RP) or late BCR(after 12 months from RP).

| Characteristic | Persistently Positive PSA  (n=1,919) | Early BCR  (n=854) | Late BCR  (n=1,598) | p-value  (PPP vs Early BCR) |
| --- | --- | --- | --- | --- |
| Age, Median (IQR) | 65 (60, 69) | 65 (59, 69) | 65 (60, 69) | 0.5 |
| Race |  |  |  | 0.003 |
| White | 1,361 (71%) | 656 (77%) | 1,254 (78%) |  |
| African American | 296 (15%) | 120 (14%) | 181 (11%) |  |
| Other | 49 (2.6%) | 18 (2.1%) | 33 (2.1%) |  |
| Unknown | 213 (11%) | 60 (7.0%) | 130 (8.1%) |  |
| Charlson Score |  |  |  | 0.7 |
| 0 | 1,381 (72%) | 611 (72%) | 1,192 (75%) |  |
| 1 | 344 (18%) | 148 (17%) | 248 (16%) |  |
| ≥2 | 193 (10%) | 95 (11%) | 158 (9.9%) |  |
| BMI, Median (IQR) | 28.9 (25.8, 32.3) | 29.0 (26.0, 32.5) | 28.8 (26.2, 32.1) | 0.4 |
| Clinical T-Stage |  |  | <0.001 | 0.3 |
| cT1 | 1,054 (55%) | 491 (58%) | 1,015 (64%) |  |
| cT2 | 585 (31%) | 250 (29%) | 449 (28%) |  |
| cT3 | 75 (3.9%) | 23 (2.7%) | 21 (1.3%) |  |
| cTx | 197 (10%) | 87 (10%) | 111 (7.0%) |  |
| Pre-Operative PSA (IQR) | 10 (6, 17) | 8 (6, 13) | 7 (5, 10) | <0.001 |
| Pre-Operative PSA Group |  |  | <0.001 | <0.001 |
| <10 | 981 (52%) | 521 (62%) | 1,144 (73%) |  |
| 10-20 | 538 (29%) | 222 (26%) | 330 (21%) |  |
| 20-50 | 283 (15%) | 82 (9.7%) | 88 (5.6%) |  |
| >50 | 77 (4.1%) | 17 (2.0%) | 13 (0.8%) |  |
| Biopsy ISUP GG |  |  | <0.001 | <0.001 |
| GG1 | 145 (7.6%) | 61 (7.2%) | 157 (9.9%) |  |
| GG2 | 437 (23%) | 259 (30%) | 584 (37%) |  |
| GG3 | 562 (29%) | 241 (28%) | 451 (28%) |  |
| GG4 | 411 (22%) | 161 (19%) | 266 (17%) |  |
| GG5 | 354 (19%) | 128 (15%) | 135 (8.5%) |  |
| NCCN Risk Group |  |  | <0.001 | <0.001 |
| Very Low | 13 (0.7%) | 6 (0.7%) | 14 (0.9%) |  |
| Low | 95 (5.0%) | 38 (4.5%) | 109 (6.8%) |  |
| Favorable Intermediate | 183 (9.6%) | 103 (12%) | 288 (18%) |  |
| Unfavorable Intermediate | 676 (35%) | 351 (41%) | 733 (46%) |  |
| High | 335 (18%) | 126 (15%) | 168 (11%) |  |
| Very High | 608 (32%) | 226 (27%) | 281 (18%) |  |
| Prostatectomy Year Category |  |  |  | 0.2 |
| Pre-2020 | 1,144 (62%) | 558 (66%) | 1,156 (72%) |  |
| 2020 | 134 (7.3%) | 61 (7.2%) | 177 (11%) |  |
| Post-2020 | 554 (30%) | 231 (27%) | 265 (17%) |  |
| Prostatectomy GG |  |  |  | <0.001 |
| GG1 | 63 (3.3%) | 23 (2.7%) | 64 (4.0%) |  |
| GG2 | 459 (24%) | 263 (31%) | 687 (43%) |  |
| GG3 | 630 (33%) | 299 (35%) | 542 (34%) |  |
| GG4 | 219 (11%) | 66 (7.8%) | 123 (7.7%) |  |
| GG5 | 538 (28%) | 196 (23%) | 173 (11%) |  |
| Prostatectomy T-Stage |  |  |  | <0.001 |
| pT2 | 521 (27%) | 296 (35%) | 797 (50%) |  |
| pT3a | 674 (35%) | 341 (40%) | 565 (35%) |  |
| pT3b | 694 (36%) | 214 (25%) | 234 (15%) |  |
| pT4 | 27 (1.4%) | 3 (0.4%) | 1 (<0.1%) |  |
| Prostatectomy N-Stage |  |  |  | <0.001 |
| pN0 | 1,337 (70%) | 683 (80%) | 1,289 (81%) |  |
| pN1 | 351 (18%) | 67 (7.8%) | 61 (3.8%) |  |
| pNx | 231 (12%) | 104 (12%) | 248 (16%) |  |
| Positive Surgical Margins | 1,124 (59%) | 511 (60%) | 704 (44%) | 0.5 |
| Adverse Pathology (GG 4/5 and T-Stage 3/4) | 662 (34%) | 205 (24%) | 194 (12%) | <0.001 |

Abbreviations: RP, radical prostatectomy; PPP, post-RP PSA persistence; BCR, biochemical recurrence; IQR, interquartile range; GG, grade group
